# Breaking Beyond the Borders of the Brain: Self-Control as a Situated Ability

**DOI:** 10.3389/fpsyg.2021.617434

**Published:** 2021-06-03

**Authors:** Jumana Yahya

**Affiliations:** Institute of Cognitive Science, University of Osnabrück, Osnabrück, Germany

**Keywords:** self-regulation, synchronic self-control, situated cognition, situated self-control, intracranialism, embodied self-control, extended self-control, distributed self-control

## Abstract

“I just couldn’t control myself” are the infamous last words of a person that did something that they knew they should not have done. Consistent self-control is difficult to achieve, but it is also instrumental in achieving ambitious goals. Traditionally, the key to self-control has been assumed to reside in the brain. Recently, an alternative has come to light through the emergence of *situated theories of self-control*, which emphasize the causal role of specific situated factors in producing successful self-control. Some clinical interventions for motivational or impulse control disorders also incorporate certain situated factors in therapeutic practices. Despite remaining a minority, situated views and practices based on these theories have planted the seeds of a paradigm shift in the self-control literature, moving away from the idea that self-control is an ability limited to the borders of the brain. The goal of this paper is to further motivate this paradigm shift by arguing that certain situated factors show strong promise as genuine causes of successful self-control, but this potential role is too often neglected by theorists and empirical researchers. I will present empirical evidence which suggests that three specific situated factors – clenched muscles, calming or anxiety-inducing environmental cues, and social trust – exhibit a specialized effect of increasing the likelihood of successful self-control. Adopting this situated view of the ability to regulate oneself works to reinforce and emphasize the emerging trend to design therapies based on situated cognition, makes self-control more accessible and less overwhelming for laypeople and those who struggle with impulse control disorders, and opens a new avenue of empirical investigation.

## Introduction

“I just couldn’t control myself” are the infamous last words of a person that did something that they knew they should not have done. It is exceedingly difficult to be self-controlled, especially when there are counterproductive temptations around every corner, and often, being in control of oneself is simply *too* difficult. However, for those who are capable of being consistently self-controlled, the rewards to be reaped are priceless. Self-control is instrumental for achieving ambitious goals and those people who have mastered this ability are more successful in school (Mischel et al., 1989; Duckworth and Seligman, 2005), are better at regulating emotions (Boden and Thompson, 2015), are more likely of having a healthy body mass index (Schlam et al., 2013), are better at coping with social rejection (Ayduk et al., 2000), and are overall happier (Hofmann et al., 2014).

The impressive benefits of being self-controlled have created a demand for understanding the nature of this ability and how it can be exercised in the right sorts of ways. Traditionally, the key to understanding self-control has been assumed to reside in the brain, as evident by the persistent habit of self-control theorists constricting their scope of investigation to cognitive and neural processes. Factors that are external to the brain, such as bodily states, environmental cues, and social interactions receive a minority of attention regarding the (potential) causal roles that they play in how a self-control dilemma unfolds. However, the ever-growing number of impulse control disorders indicate that perhaps the current popular strategies for increasing self-control are not so effective and efficient.

Recently, an alternative has come to light through the emergence of several theories of self-control that go against what has become a core assumption for much of the literature: the brain is the cause of self-control. These views have their roots in situated cognition, the view that cognition depends on not only the brain, but also upon certain situated factors, including bodily states, environmental cues, and/or social interactions (Walter, 2014). Such *situated theories of self-control* emphasize the causal role of specific situated factors in producing successful self-control (e.g., Balcetis and Cole, 2009; Heath and Anderson, 2010; Vierkant, 2014). Some clinical interventions for motivational or impulse control disorders also incorporate certain situated factors in therapeutic practices, such as the focus on bodily states in mindful meditation as a therapy for addiction (Black, 2014), aggression (Singh et al., 2007), and post-traumatic stress disorder (King et al., 2013), or the focus on environmental cues in sensory rooms used to treat apathy in dementia patients (Staal et al., 2007). Despite remaining a minority, situated views, as well as the practices based on these views, have planted the seeds of a paradigm shift in the self-control literature, moving away from the idea that self-control is an ability limited to the borders of the brain.

The goal of this paper is to further motivate this paradigm shift by arguing that certain situated factors show a lot promise as genuine causes of successful self-control, but this potential role is too often neglected by theorists and empirical researchers. In order to do so, I will explain the source of contention between “traditional” and situated self-control theories in section “Setting the Stage.” Then, in section “Empirical Evidence for Situated Self-Control,” I will present empirical evidence which suggests that three specific situated factors – clenched muscles, calming or anxiety-inducing environmental cues, and social trust – exhibit a specialized effect of increasing the likelihood of successful self-control. Lastly, in section “Taking Stock and Moving Forward,” I will take stock of the situation by briefly discussing certain implications of taking the position that self-control is situated. Adopting this view works to reinforce and emphasize the emerging trend to design therapies based on situated cognition, makes self-control more accessible and less overwhelming for laypeople and those who struggle with impulse control disorders, and opens a new avenue of empirical investigation.

## Setting the Stage

Theories, debates, and research pertaining to the nature of self-control comprise a large body of literature that has an extensive history and many interdisciplinary contributions. This variety contributes to the complexity and density of self-control as a concept. Similarly, situated cognition, albeit being an incredibly young concept relative to self-control, is also quite complex and dense. In order to smoothly navigate the conceptual merger between self-control and situated cognition, it is useful to review some fundamental definitions, distinctions, and terms.

In this section, I will clarify important terms and concepts that are liberally referenced throughout the remainder of the paper. First, I will provide a definition of self-control and explain certain important distinctions that are relevant to the arguments in the next section. Then, I will discuss the basic role of the brain, as well as offer some suggestions as to why the brain is assumed to bear so much of the causal burden for successful exercises of self-control. Lastly, I will present the general idea of situated cognition and how it applies to self-control.

### Self-Control

It is quite an onerous task to develop a universal definition of self-control that most theorists accept without hesitation or resistance. There is a considerable amount of variation – across disciplines, as well as within – on how to define self-control^1^. For the sake of being as inclusive of the various views as possible, I will adopt the following generally broad definition:

SELF-CONTROL refers to the ability to regulate one’s own thoughts, emotions, and behaviors for the sake of achieving a particular goal(s), especially when motivational opposition is present.

This definition, albeit being non-controversial^2^, is nevertheless conceptually dense and requires some further clarification before we can move on to the arguments regarding whether the brain alone is responsible for exercising this ability.

A good place to begin unpacking the proposed definition is by explaining the *function* of regulating oneself. Self-control is instrumental in achieving one’s goal(s). A goal can range from being concrete and extremely specific (e.g., “I want to lose 50 pounds by Christmas”) to being abstract and vague (e.g., “I want to have an attractive body”). A goal can also range from being achievable in a relatively short span of time (e.g., taking an Introduction to Business course, which takes one semester) or it can require a more long-term commitment (e.g., pursuing a Master of Business degree, which takes several years). The goals which people usually care about the most are those which may be called “higher aspirations,” such as improving one’s social status, becoming wealthy, eliminating bad habits, being an effective leader for a large group of people (either in a professional project or in a social movement), or mastering a complex skill. Such goals tend to be more difficult to achieve than more basic desires like simply maintaining one’s social status and current income, avoiding extra responsibilities, and being able to make do with the skills that one already possesses. Higher aspirations are usually formulated in an abstract or vague way (e.g., “I want to be wealthy” instead of “I want to receive a gross income of five million dollars a year by age 40”), which makes it difficult to know exactly what needs to be done in order to achieve the goal, and they are almost always long-term goals, which require extra dedication and resources to see through until the end. While self-control can certainly be useful for achieving the more concrete and short-term goals, this ability is especially beneficial for achieving the more abstract and long-term goals, as these are much more susceptible to being threatened by some form of *motivational opposition*.

Motivational opposition occurs when an agent has some reason(s) for acting in a way that is contrary to or impedes her goal(s), such as when an individual who is on a strict sugar-free diet experiences the desire to indulge in a large slice of chocolate cake. Motivational opposition also includes instances that involve some reason(s) to refrain from acting altogether, like, for example, when a very lazy individual who is passively lounging in bed has an important deadline but will not muster the energy to get up and start working^3^. When an agent experiences motivational opposition, she faces a *self-control dilemma*: she must choose between the difficult and unpleasant task of resisting the opposition, which is likely to lead to the best ultimate outcome, or she can take the easy road of succumbing to the opposition, which might feel good at the moment but will very likely lead to undesirable consequences. The agent must recognize and acknowledge that there will be negative consequences of succumbing to the opposition while, at the same time, still feeling a stronger motivational pull to succumb, as this is the crux of the dilemma^4^.

To sum up thus far: the general function of self-control is to facilitate the achievement of goals, especially those goals which are more abstract and/or long-term, which also happen to be the goals which we typically care about most and hence have a strong desire to pursue. Self-control is needed in order to achieve these goals because they are vulnerable to threats by motivational opposition (i.e., the desire to do something else or the lack of desire to do the thing one is supposed to do). When opposition arises, the agent faces a self-control dilemma and must choose between resisting the opposition, which is harder but will likely result in ultimately better consequences, or succumbing to the opposition, which is easier but will likely result in ultimately worse consequences.

Succumbing to the motivational opposition with little to no resistance is essentially *weakness of will*, that is, intentionally acting contrary to one’s goal(s) (McIntyre, 2006). An agent who instead chooses to resist the opposition is not necessarily self-controlled, as her efforts can either fail or succeed. An agent who tries to resist the opposition but ends up acting in a way that impedes her goal – such as the dieter who fights her craving for the chocolate cake but ends up caving into the desire by indulging in a slice – illustrates an instance of a *self-control failure*. *Successful self-control*, on the other hand, occurs when an attempt to resist some motivational opposition results in the relevant cognitive change such that the agent’s corresponding behavior either promotes or, at the very least, does not impede her goal(s). Strategies or interventions which are intended to help those who are facing a self-control dilemma ideally work to increase the likelihood of successful self-control. Many philosophers who are concerned with self-control debates focus on the relationship between weakness of will and self-control by asking such questions as “how is it possible to intentionally resist some powerful temptation, which is the thing you want most right now?” (e.g., Mele, 1992; Kennett and Smith, 1996)^5^.

I will take it for granted that intentional resistance against some form of motivational opposition is possible, and instead will discuss accounts of how *successful* self-control is possible. More specifically, this paper is concerned with understanding the cause(s)^6^ of successfully regulating oneself and the kinds of strategies that can be implemented to ensure victory over motivational opposition. In the following part, I will explain the origin of the incredibly pervasive assumption that self-control is an ability that belongs exclusively to the brain.

### The Role of the Brain and the General  Neglect of Situated Factors 

While there is much philosophical debate about the nature of self-control, there is significant consensus about the psychology and neurology of self-control amongst researchers and medical professionals. There are certain empirical observations regarding the importance of mindset which lead researchers to draw a connection between self-control and the brain, but emphasis of this connection likely leads to the neglect toward considering the potential role of situated factors in self-control.

There is considerable evidence that a particular cognitive state, or mindset, works to significantly increase the likelihood of successful self-control. This mindset is comprised of several related beliefs and feelings: that one is autonomous and competent (Ryan and Deci, 2000), that one’s attributes are malleable rather than fixed (Burnette et al., 2013), confidence and affirmation of one’s own worth (Vandellen et al., 2012), pride in one’s own achievements (Tracy, 2016), and passionate determination to persevere in the face of challenges (Duckworth and Quinn, 2009); these various cognitive states contribute to one’s perception of self, specifically pertaining to themes such as strength, control, and power. Taken together, these studies indicate that a specific mindset, namely, the affirmation of one’s own strength, control, and power significantly increases the likelihood of successful self-control.

Based on the suggestion that a specific mindset can cause self-control, a quite common prescription for increasing self-control is to manipulate certain cognitive states; the idea is that changing the thought process changes the behavior. Consequently, the most common sorts of strategies for increasing the likelihood of self-control involve mental actions such as shifting attention (e.g., Mischel, 2014) or inhibiting recalcitrant desires (e.g., Sripada, 2014). The persistence of prescribing self-controlling strategies that consistently require some form of mental gymnastics – that is, consciously effortful mental feats – with no suggestions of how to manipulate certain bodily, environmental, or social factors is a strong indicator that the design of such strategies reflects a bias where the potential direct impact of situated factors on the success of self-control is significantly neglected.

Strategies involving shifting attention or inhibiting recalcitrant desires often recruit certain mental functions, like executive attention, inhibition, or working memory. These mental functions are correlated with certain neural areas, which happen to be located within the prefrontal cortex; the brain area that is perhaps the most associated with self-control is the ventromedial prefrontal cortex, including areas such as the orbitofrontal cortex, the lateral prefrontal cortex, and the anterior cingulate cortex (Heatherton, 2011). The relationship between the mental functions recruited for self-control and the neural correlates with which they are associated is further reinforced by the success of certain approaches that incorporate neural activity as an integral part of the therapy, such as measuring activity in the lateral prefrontal cortex to gauge cognitive training of proactive cognitive control (Berkman et al., 2014), or using amygdala activity to help implement attention bias modification to attenuate anxiety (Britton et al., 2014). So, while there certainly seems to be some sort of connection between self-control and the brain, the complexity of the brain makes it quite difficult to explain exactly what this connection amounts to (Berkman, 2018) and any tentative conclusions about this connection should be treated with caution. The emphasis that many self-control theorists place on the brain within their discussions of how it is possible to exercise this ability (e.g., Knoch and Fehr, 2007) threatens to further perpetuate negligence toward the potential role that situated factors play.

For the sake of both clarity and ease, I will call views that assume that self-control is caused only by the brain “intracranialist” positions since such views constrict this ability to the confines of the cranium. Furthermore, when I reference “the brain” or “brain-based strategies,” I am referring to the cognitive processes that are consciously recruited for self-control or the strategies that rely exclusively on these processes. In the next part, I will explain the fundamental differences between a situated and an intracranialist view of self-control.

### Situated Self-Control

Situated cognition is an umbrella concept which denotes any view that the mind is not constricted to the borders of the brain, but also involves some situated factors (e.g., bodily states, environmental cues, and/or social interactions) as either a *cause* or a *constituent* of cognition (Clark and Chalmers, 1998; Walter, 2014). The term situated is used very broadly and comes in many different flavors. Situated cognition includes any theories relating to the mind that can be called *embodied* (i.e., emphasis on either the causal or constituent relation between cognition and bodily states), *embedded* (i.e., emphasis on the causal relation between cognition and environmental cues), *extended* (i.e., emphasis on the constituent relation between cognition and environmental cues), *enacted* (i.e., emphasis on sense-making through interactions between bodily states, environmental cues, and social interactions), or *distributed* (i.e., emphasis on the relation between cognition and social interactions) (Walter, 2014). Situated cognition is a concept directly opposing that of intracranialism, or the view that the brain alone is responsible for cognition, which has been the dominant assumption within the cognitive sciences. Some have applied the concept of situated cognition to specific cognitive states and processes, affectivity being currently the most popular (e.g., Fuchs and Koch, 2014; Colombetti and Krueger, 2015; Colombetti, 2017). Stephan et al. (2014) nicely encompass this paradigmatic pivot with a single question: is it possible that “the brain alone can do some emoting?” One can probably pose this question for an array of different cognitive states, including cognitive abilities like self-control. When considering whether the brain alone can do some *self-controlling*, a handful of situated theories of self-control have emerged (e.g., Balcetis and Cole, 2009; Heath and Anderson, 2010; Hung and Labroo, 2011; Vierkant, 2014). Situated theories of self-control show promise for evolving our understanding and knowledge of self-control, and the practical implications alone – in terms of designing alternative therapies for disorders of the self (Krueger and Colombetti, 2018) – should be sufficient for these views to gain significant attention. Considering that such views, unfortunately, remain the minority within the literature, it becomes important to seriously revisit this question: “can the brain alone do some self-controlling?”

The answer, as it turns out, is a bit complicated. If we take “doing some self-controlling” as ascribing causal responsibility, then one can defend a variety of claims. One can take an extreme position and argue that either the brain alone or situated factors alone can have any sort of impact on self-control. It is also possible to take a weaker position and argue that both the brain and situated factors have an impact on self-control, but the kind of impact can vary. In order to explain this distinction between different kinds of impact, I will use the word *cause* to refer to a thing that directly and consistently produces an effect, and *influence* to refer to a thing which that facilitates an effect, simply by making the surrounding conditions more favorable for the effect to take place (c.f. Sripada, 2014 for similar distinction). Considering the variety of claims that each position can defend highlights that the fundamental difference between some specific intracranialist and situated views regarding self-control can be quite nuanced. Below are five substantially different claims that can be defended by either an intracranialist or situated view of self-control:

(1) The brain causes self-control.
(2) The brain causes self-control, although situated factors can have an influence.
(3) The brain and situated factors both cause self-control.
(4) Situated factors cause self-control, although the brain could have an influence.
(5) Situated factors cause self-control.

Claims (1) and (5) represent the two most extreme positions that one can take regarding the cause of successful self-control. Claim (2) is a weaker version of an intracranialist view, whereas claim (4) is a weaker version of a situated view. It is more accurate to identify claim (3) as a situated position since ascribing causal responsibility to something outside of the cranium acts as a counterexample to intracranialism. In other words, endorsing claim (3), and thus also admitting that certain situated factors have as much causal responsibility as the brain, is incompatible with the core assumption that self-control operates only within the borders of the cranium. For these reasons, claims (1) – (5) can be assigned the following positions:

#### Intracranialist Views of Self-Control

(STRONG) The brain causes self-control.
(WEAK) The brain causes self-control, although situated factors can have an influence.

#### Situated Views of Self-Control

(WEAK) The brain and situated factors both cause self-control.
(INTERMEDIATE) Situated factors cause self-control, although the brain could have an influence.
(STRONG) Situated factors cause self-control.

In the next section, I will argue in support of the weak position of situated self-control because my goal is not to denounce the role of the brain. Rather, my aim is to emphasize the role that certain situated factors play in significantly increasing the likelihood of successful self-control, to the extent that such factors ought to be considered just as an important for self-regulation as the brain.

## Empirical Evidence for Situated Self-Control

The claim that certain situated factors can cause self-control has not been explicitly tested in the thorough and rigorous way that it arguably deserves. However, there is some empirical work that can shine some light on the matter. First, it is important to have a standard set of criteria for what counts as a cause, in order to be able to systematically analyze different situated factors to see which qualify as situated causes of self-control. Having an impact on self-control is by itself an insufficient criterion because too many irrelevant factors can be included. Eating ample amounts of vitamin C, for example, leads to an energetic state, but simply having energy does not guarantee success over motivational opposition, although it certainly helps. A mere influence has a general impact on self-control, whereas a bona fide cause must satisfy stricter criteria.

In this section, I will provide empirical evidence that suggests that certain situated factors have causal power in bringing about successful self-control. First, I will present a set of studies that demonstrate the causal power of a certain bodily state and briefly discuss the criteria which the investigators adopt to identify a genuine cause of self-control; namely that the factor in question must have a *specialized effect*. Then, I will present an experiment that suggests that a particular type of environmental cue can replenish self-control resources and apply these criteria to indicate that this may also be an example of a genuine situated cause of successful self-control. Finally, I will do the same thing for an example involving a particular social cue and its potential specialized effect on delay of gratification.

### Bodily Cause Identifies Criteria

In recent years, with the surge in popularity of eastern philosophical ideas and practices, the concept of embodiment has gained quite a lot of attention. The harmony between mind and body is a central tenet in many current self-development practices, such as practicing yoga, breath-work, and mindful meditation, or meticulously planning one’s nutrition to include as much “brain food” as the body can feasibly process. The world of clinical psychology has also joined the trend by incorporating embodiment into the design of therapies, using dance, for example, to express oneself and as a therapeutic release of energy. While the concepts of embodied cognition (e.g., Pulvermueller, 2005) and embodied affectivity (e.g., Fuchs and Koch, 2014) have received significant empirical and theoretical support (as well as their fair share of criticism), embodied self-control is a concept that has been discussed only by a small minority (e.g., Balcetis and Cole, 2009). Can certain bodily states cause successful self-control?

The most direct evidence for the effect that certain bodily states have on successful self-control comes from a set of experiments that demonstrate that muscle tension (e.g., clenched fists or tightened calf muscles) significantly increases self-control in a variety of domains (Hung and Labroo, 2011). This set of studies aims to confirm that the physical expression of recruiting and firming willpower (e.g., clenching one’s fists) also works to recruit and firm willpower. The results reveal that participants who were clenching their muscles were much more successful than their relaxed counterparts at completing an array of self-control related tasks, such as being able to withstand the discomfort of attending to unwanted stimuli, drinking large amounts of a disgusting vinegar-based “health drink,” enduring physical pain for long periods of time, and making healthier food choices during snack time.

The bodily state of firming one’s muscles qualifies as a legitimate cause of self-control for two reasons: (1) instead of modulating the cognitive state which then mediates the success or failure of self-control, this bodily state has a non-conscious and *direct impact* on self-control^7^; and (2) this bodily state has a *specific impact*, in that it works only to *improve* self-control, in virtue of being “inherently tied to [strengthening or] summoning willpower” (Hung and Labroo, 2011). Creating this *specialized effect* (i.e., direct and specific impact) is a crucial criterion for identifying whether some situated factor is a cause of successful self-control^8^. If the presence of some situated factor has an effect on self-control, but this effect is indirect and/or general (e.g., affirming the belief that one is autonomous and competent so that this belief improves self-control), then it is more appropriate to characterize the situated factor in question as a mere influence rather than a bona fide cause.

Unfortunately, given how young and underdeveloped the concept of situated self-control happens to be right now, there is not much additional evidence that clearly supports a causal link between various situated factors and successful self-control. While the relationships between situated factors and cognition or affectivity have received a considerable amount of empirical attention, situated self-control has yet to receive its fair share of investigation. However, being equipped with at least one criterion for identifying these situated causes makes it easier to assess other empirical studies that are not explicitly endorsing situated self-control but are nevertheless relevant. In the following part, I will apply this criterion to an example consisting of a specific type of environmental cue that replenishes self-control resources in order to propose that certain environmental cues can also be potential causes of successful self-control.

### Candidates for (Environmental) Situated Causes of Self-Control

A person’s immediate environment contains numerous cues that can directly affect certain cognitive states, such as the smell of lavender working to decrease stress and attenuate the perception of pain (Kim et al., 2011). Features of one’s environment can also directly affect certain behaviors, like red-colored plates working to curb excessive eating (Genschow et al., 2012). If a particular environmental cue produces a specialized effect (i.e., an increase in the likelihood of successful self-control in virtue of this cue being inherently tied to strengthening willpower), then such a cue becomes an eligible candidate for being a cause of self-control. Two such eligible candidates are the calm-inducing cues found in natural environments and the anxiety-inducing cues found in urban environments.

Based on evidence that a natural environment has a restorative effect on cognitive processes (Gamble et al., 2014), one study investigates whether environment type can restore self-control resources and finds that environmental compatibility modulates this effect, in that the type of environment has to be compatible with the individual’s personality (Newman and Brucks, 2016). More specifically, natural environments have a restorative effect only for personality types low in neuroticism, whereas personality types high in neuroticism experience the same restorative effect in urban environments (Newman and Brucks, 2016). The proposed reason for this effect is that processing certain environmental cues can require less attentional resources because of a sense of familiarity between the personality type and the type of cue, therefore allowing the resources to be replenished. Individuals high in neuroticism, for example, can process complex and dynamic environmental cues with less attentional effort because such individuals are more familiar or comfortable with anxiety-associated cues. This familiarity makes engaging with urban environments require less attentional resources, thus urban environments offer opportunity to recuperate and, in that sense, could be more restorative for neurotic agents. Conversely, calming environmental cues would require more attentional resources from a neurotic agent because of the lack of familiarity – such cues would have to be processed similarly to how novel cues are processed – which would prevent a state of recuperation and replenishment of resources.

So, do these specific kinds of environmental cues have a direct and specific impact on improving self-control? Well, the impact of these cues certainly seems direct in the sense that they affect self-control (resources) directly rather than modulating the cognitive state that, in turn, modulates the likelihood of self-control. In order to determine if such cues satisfy the *specific impact* condition, these cues must work to improve self-control in virtue of being inherently tied to strengthening or summoning willpower.

Currently, there is not enough empirical data to claim, with certainty, that strengthening or summoning willpower inherently involves some type(s) of environmental cues. To my knowledge, such empirical investigation has not yet been conducted. It is nonetheless possible to speculate based on certain colloquial beliefs about the power of environments in providing certain advantages during competitions. In the world of competitive sports, for example, there is an idea known as the ‘home team advantage’, which posits that players who perform within their “home” arena, where most of their practice sessions and some of their competitions occur, are privy to an advantage over the players who are performing in this arena for the first time (Courneya and Carron, 1992; Swartz and Arce, 2014). One of the potential reasons why the home arena provides such an advantage is due to familiarity with the stable environmental cues (e.g., the layout of the arena), which makes it much easier and quicker to process the immediate environment, thus freeing up cognitive processes to focus on the matter at hand (Legaz-Arrese et al., 2013), which, in this case, is beating the competition. In this sense, certain environmental cues that comprise the “battle arena” can be construed as being inherently tied to the battle itself, such that changes in the arena directly impact the performance. While it is obvious what a home arena is within the context of sports competitions, it is much less obvious what would comprise a home arena – an environment that provides a competitive advantage based on familiarity – in a self-control dilemma. However, the concept of a home team advantage reflects the concept of environmental compatibility highlighted by Newman and Brucks (2016), in that familiarity with a specific type of cue (i.e., anxiety-inducing or calming) provides an advantage for self-control, namely, self-control resources being replenished. Following the analogy of a sports competition, it is plausible that environmental compatibility provides an advantage for an agent who is facing a self-control dilemma due to the inherent relationship between the arena (i.e., whether the agent is in an environment which contains calming or anxiety-inducing cues) and the agent herself (i.e., whether she is low or high in neuroticism, respectively).

Newman and Brucks (2016) demonstrate that calm/anxiety-inducing cues work to replenish self-control resources when the type of cue is compatible with the personality type of the agent. This observation by itself might not provide direct evidence that these specific types of cues are situated causes of self-control, since whether these cues exhibit a *specific* impact (i.e., improve self-control in virtue of being inherently tied to strengthening/summoning willpower) has not (yet) been explicitly investigated. However, as previously mentioned, the lack of explicit empirical evidence may well be due to a general lack of diligent investigation. The aim here is not to provide a convincing argument that calming/anxiety-inducing cues are undoubtedly situated causes of successful self-control, but rather to show the plausibility of identifying bona fide situated causes of successful self-control by sharing empirical evidence that strongly hints in this theoretical direction. Further empirical corroboration is needed in order to establish these cues, or other qualifying environmental factors, as situated causes of self-control. In the following part, I will discuss a certain social cue that appears to be important for an individual’s willingness to delay her gratification and argue that this social cue is another plausible situated cause of successful self-control.

### Candidate for (Social) Situated Cause of Self-Control

High achievers often cite the quality and depth of their social networks as one of the keys to their success. The idea that social support is a powerful tool is a key tenet of addiction recovery groups such as Alcoholics Anonymous and Narcotics Anonymous. In our modern world, people can become millionaires simply by building communities on social media platforms like Instagram, YouTube, and Facebook. It is difficult to deny the powerful effect of social factors on cognition and behavior, but can such factors qualify as genuine causes of successful self-control?

There is at least one social factor that appears to be an eligible candidate for being a situated cause of self-control: trust. Two related experiments reveal that impressions of trustworthiness affect the willingness to delay gratification (Michaelson et al., 2013). Participants were presented with vignettes and pictures of characters that vary in implicit trustworthiness (e.g., pictures of people exhibiting “untrustworthy” facial expressions) and then placed in a classic (hypothetical) delay of gratification scenario (i.e., given a choice between an immediate smaller reward or a later larger reward) with those same characters. Participants who were paired with untrustworthy characters were more likely to choose the lesser but more certain reward, whereas those paired with trustworthy characters were significantly more willing to delay their instant gratification in exchange for the larger later reward. A follow-up experiment confirmed that trust has this impact on the willingness to delay gratification irrespective of other relevant factors, such as exerting cognitive effort to regulate oneself or intentionally modulating the perception of reward (Michaelson et al., 2013).

The impression of trustworthiness has a direct impact on self-control, and this social factor works to specifically *improve* self-control by increasing the willingness to delay gratification. In considering whether trustworthiness qualifies as a situated cause of successful self-control, the question which remains to be answered asks whether trustworthiness is inherently tied to strengthening or summoning willpower. Just as with the case of environmental cues, there is yet to be conclusive evidence that trustworthiness is inherently tied to strengthening or summoning willpower, but it is possible to speculate.

While many instances of self-control dilemmas are experienced privately and thus do not contain a social dimension, it can be argued that all instances of a self-control dilemma necessarily involve trustworthiness. A key premise for such an argument is that trustworthiness applies not just to (other) social being, but also to certain non-social factors such as one’s immediate environment. For example, Krueger and Colombetti (2018) argue that trustworthy access to certain affordances provided by one’s immediate environment is crucial for the regulation of affective states. Take, for example, an affordance provided by the whiteboard hanging in my office, namely, that I can use this board to write down important reminders and thus not worry about constantly keeping this information in my working memory. For this information to have an impact on my behavior (e.g., sitting down in front of my phone because it is written on the whiteboard that I have a call meeting coming up), I must trust the information written on this board. If, to push the example further, I bought this whiteboard at a joke shop and I know that any memos I write to myself are not reliable because the whiteboard changes numbers that are written on it, then seeing a call meeting reminder for a specific time written on this board will *not* motivate me to take out my phone and prepare for a meeting. Similarly, if I accidentally purchase the prank whiteboard thinking it is an average whiteboard, then I will have no reason to doubt the reliability of what is written on that board and I will sit down for my meeting at the wrong time. The point here is that throughout the different variations of this scenario, my behavior – what time I sit down to prepare for my meeting – is highly dependent on whether I perceive the whiteboard as a reliable reminder. When I trust the whiteboard, the memos correspondingly affect my behavior, but not when I perceive the whiteboard to be untrustworthy. Trustworthiness, therefore, does not necessarily apply to only people, and could very well be a factor inherently tied to strengthening or summoning willpower.

To reiterate once more, this speculation of how trust might inherently be tied with strengthening or summoning willpower is not meant to be undoubtedly convincing. Instead, the aim of this section is to suggest a viable candidate that has been shown to have a direct impact on self-control. The more viable candidates that are proposed, the more motivation there is for a paradigmatic shift of focus toward being more diligent and serious about considering situated factors as potential causes of successful self-control.

## Taking Stock and Moving Forward

I have presented evidence that supports the claim that self-control is situated, in that certain situated factors have a direct and specific impact on improving self-control in virtue of being inherently tied to strengthening or summoning willpower. Studies that support the causal power of three situated factors (i.e., bodily state, environmental cue, and social cue) were discussed as potentially demonstrating examples of situated causes of self-control. The first set of experiments present explicit evidence for the causal power that clenched muscles exhibit over successful self-control (Hung and Labroo, 2011).

Another study provides evidence that the presence of calming or anxiety-inducing cues works to replenish self-control resources for non-neurotic or highly neurotic individuals, respectively. This study reveals a direct impact of such cues on self-control resources but does not investigate whether these cues have a specific impact of improving self-control in virtue of the cues being inherently tied to strengthening or summoning willpower. I provided some intuitive speculations of how such an inherent relationship could plausibly exist, but further empirical testing is required to establish that such a relationship indeed exists. Similarly, two related studies reveal that impressions of trustworthiness directly impact the willingness to delay gratification, but the researchers do not offer any arguments as to whether trust is inherently tied to strengthening and summoning willpower. I provided some speculations on this point as well. Admittedly, only the first example explicitly shows some situated factor exhibiting a specific impact of improving self-control in virtue of being inherently tied with strengthening or summoning willpower, but the other two examples reveal, at the very least, viable and promising candidates.

Although there is currently no demonstrative proof that these two situated factors are inherently tied with willpower, a plausible and empirically verifiable story can be told, thus contributing to the viability of their candidacy for being considered bona fide causes of self-control. It is not very surprising that older theories did not consider the potential role of situated factors in producing successful self-control, given how the popularity of situated cognition is relatively new. What is surprising, however, is how little attention the concept of situated self-control has received in contemporary research compared to much indication there is that this would be a worthwhile empirical and philosophical investigation.

One major practical benefit of unburdening the brain of sole causal responsibility for successful self-control is that exercising this ability becomes exponentially easier. Since situated causes operate non-consciously and in a reflexive-like way, the result can be achieved without conscious effort, and not having to intentionally invest conscious effort greatly reduces – if not eliminates altogether – feelings of struggle or difficulty. Delegating the work of regulating oneself to non-conscious processes thus creates an “effortless” experience. Since the anticipation of struggle or difficulty is what causes many people who face a self-control dilemma to feel too overwhelmed to attempt being self-controlled (Milyavskaya and Inzlicht, 2017), a less effortful experience can circumvent this consequence.

The goal of this paper is to make the case that empirical research concerned with self-control, as well as therapeutic interventions that are designed to treat impulse control disorders, will greatly benefit from abandoning the idea that the brain alone is causally responsible for successful self-controlling. Currently, some situated theories of self-control have already been offered and there have even been some experimental interventions that rely on situated factors to provide therapeutic benefits. However, such theories and therapies should no longer be just interesting alternatives and deserve much more theoretical and empirical attention than they have thus far received. There is so much potential for creativity, growth, and innovation for the interdisciplinary field of self-control research, and this full potential can be unleashed by simply breaking beyond the borders of the brain.

## Author Contributions

JY contributed equally to the writing and editing of this article.

## Conflict of Interest

The author declares that the research was conducted in the absence of any commercial or financial relationships that could be construed as a potential conflict of interest.

## Footnotes

1 Some of these differences are due to the fact that many self-control theorists draw a distinction between self-control and other related concepts such as willpower (Holton, 2003) and self-regulation (Fujita et al., 2018), while other theorists conceptually consolidate these into one basic ability (e.g., Sripada, 2014). I do not make these distinctions and will use these terms interchangeably.

2 In the sense that this definition is unlikely to be accused of being too restrictive of what kinds of strategies can count as self-control (c.f. Sripada, 2014 or Fujita, 2011).

3 There are at least three different types of motivational opposition that are interchangeably discussed in the self-control literature: temptation, procrastination, and diminished motivation. Temptation refers to a competing desire, such as when an ex-smoker experiences a craving for a cigarette despite her goal to remain smoke-free. Procrastination involves a delay in pursuing one’s goal, like the classic example of a college student who waits until the last minute to start her assignment even though she wants to receive a high grade in the class. Lastly, diminished motivation refers to a lack of the desire to do anything at all, including pursuing one’s goals (Connor, 2013). An example of diminished motivation is clinical apathy or depression, which renders a person generally incapacitated even though a patient with this disorder can express the desire to get out of bed and live their life. In order to be inclusive, I will continue using the term “motivational opposition” to refer to all three types instead of constricting the discussions to only one type.

4 If the agent feels a stronger motivational pull to do something that impedes her current goal(s) but does NOT recognize any negative consequences in doing so, then she is in a position to simply update her decision or revise her goal(s) regarding what is the best thing for her to do. For example, imagine a person who aims to be a vegan feels a strong desire to indulge in a juicy piece of steak, but, at the same time, she does *not* recognize or acknowledge any negative consequences of eating the meat (e.g., she does not think meat farming is unethical; or she does not believe that eating meat is bad for her health in any way). We would expect such a person to either drop being vegan as one of her goals, as she evidently does not have any reason(s) motivating her to be vegan, or to start acknowledging some negative consequence of eating animal products.

5 This question is based on *the puzzle of synchronic self-control*. While attempting to solve this puzzle is outside of the scope of this paper, it is important to mention because this puzzle inspired many prominent theories of self-control. For an exact formulation of the puzzle and the debates that have arisen from attempted answers, see Sripada (2014) or Connor (2013).

6 I clarify what I mean by *cause* in later sections.

7 This point is corroborated especially by the second experiment in the set, where participants had to endure physical pain and those who were clenching their fists endured the pain for significantly longer than those whose fingers were kept loose and relaxed; the researchers also manipulated for belief modulation and found that clenching fists did NOT modulate any beliefs or self-perceptions, showing that firmed muscles have a direct impact on self-control.

8 Importantly, since a cause of self-control works to increase the likelihood of successful self-control by having a direct and specific impact on successful self-control, then the brain (rightfully) qualifies as a cause of successful self-control.
